# Predictive Factors of Functional Outcomes and Quality of Life in Patients with Ankle Fractures: A Systematic Review

**DOI:** 10.3390/jcm13051188

**Published:** 2024-02-20

**Authors:** Alejandro Lorente, Leire Pelaz, Pablo Palacios, María Benlloch, José Enrique de la Rubia Ortí, Carlos Barrios, Gonzalo Mariscal, Rafael Lorente

**Affiliations:** 1 Ankle and Foot Surgery Unit, Department of Traumatology and Orthopaedic Surgery, University Hospital Ramón y Cajal, 28034 Madrid, Spain; alejandro.lorentegomez@gmail.com (A.L.); dra_lire@hotmail.com (L.P.); 2 Department of Traumatology and Orthopaedic Surgery, Sanchinarro University Hospital, 28050 Madrid, Spain; pablopalacios@drpalacios.com; 3 Department of Basic Medical Sciences, Catholic University of Valencia, 46001 Valencia, Spain; maria.benlloch@ucv.es (M.B.); joseenrique.delarubi@ucv.es (J.E.d.l.R.O.); 4 Institute for Research on Musculoskeletal Disorders, School of Medicine, Valencia Catholic University, 46001 Valencia, Spain; carlos.barrios@ucv.es; 5 Department of Orthopedic Surgery and Traumatology, University Hospital of Badajoz, 06006 Badajoz, Spain; rafaalelorente@hotmail.com

**Keywords:** ankle fracture, risk factors, quality of life, functionality

## Abstract

**Background**: Evaluating the predictors of unfavorable outcomes in patients with ankle fractures is crucial for identifying high-risk patients and implementing personalized treatment strategies. This study aimed to analyze factors that influence quality of life in patients with ankle fractures. **Methods**: Four databases were consulted. The main outcomes were functionality and quality of life scales combined using the standard mean difference (SMD) (Review Manager 5.4). **Results**: Eight studies with 2486 patients were included. A significant correlation was found between female sex and worse functionality scores (beta 4.15, 95% CI 1.84–6.46). Additionally, older age was correlated with worse functionality scores (beta −0.24, 95% CI −0.29 to −0.19). Patients with diabetes or metabolic syndrome also had worse outcomes (SMD 0.27, 95% CI 0.18–0.36). High BMI and obesity were also associated with worse quality of life scores (beta 2.62, 95% CI 0.77–4.48). Smokers had greater disability in the analyzed scales (SMD 0.22, 95% CI 0.05–0.39). No significant differences were observed with respect to syndesmotic involvement. **Conclusions**: Age, sex, diabetes, high BMI, and smoking negatively impact functional outcomes and quality of life in patients with ankle fractures.

## 1. Introduction

Ankle fracture is a prevalent injury affecting a significant number of individuals worldwide. In fact, they are the fifth most common injury in the human body [1]. In the United States, ankle fractures have an estimated incidence of 4.22/10,000 person-years, and this incidence is increasing. The average age of patients affected by this injury is 37 years [2,3]. Young patients with ankle fractures may experience high indirect costs due to the need for time off and difficulty in returning to their previous activity levels, which is exacerbated by the most feared long-term complication, such as post-traumatic osteoarthritis [4].

Shah et al. underscore that, notwithstanding surgical fixation of ankle fractures, a substantial portion of patients continue experiencing enduring functional limitations even five years post-operation. Beyond this timeframe, 63% still report joint stiffness, 45% encounter ankle swelling, 50% note persistent pain, 39% perceive incomplete recovery, and 38% have not resumed their pre-injury sporting activities [5]. Furthermore, an additional retrospective longitudinal study involving 1000 patients undergoing ankle fracture fixation between 2006 and 2015 emphasizes that specific patient characteristics such as female gender, obesity, tobacco use, and alcohol consumption exert a more pronounced impact on patient-reported functional outcomes than injury characteristics, elucidating the influence of immutable risk factors on patient-reported outcome measures (PROMs) [6]. Notably, current smokers particularly exhibit an augmented predisposition to utilize prescription pain medications and display inferior patient-reported functional outcomes following surgical intervention for ankle fractures when compared to former smokers and non-smokers [7]. De las Heras et al. articulate age, occupation, cause of injury, syndesmotic lesion, number of fractured malleoli, and surgical timing delays as indispensable predictors of ultimate clinical outcomes [8]. It is important to review risk factors with the highest level of evidence to discuss the actual expectations of the patient. The primary goal of this study was to evaluate the factors that predict functional outcomes in patients with ankle fractures.

## 2. Materials and Methods

### 2.1. Elegibility Criteria

This meta-analysis registered the protocol in PROSPERO (CRD42023445384). The PRISMA guidelines [9] were followed, and the PICOS search strategy was employed as follows: (P) patients with ankle fractures who underwent surgery or conservative treatment; (I) the intervention group consisted of patients with each of the predictors of functionality or quality of life; (C) the comparative group included patients without such predictors or those with the opposite predictors; (O) the primary outcomes were functionality, quality of life, and pain scales; and (S) the included studies were comparative cohort or case–control studies. Studies that did not share the variables of interest were excluded because their results would not be homogeneous or combinable. Those with significant clinical differences that could not be statistically controlled were also excluded to avoid bias from uncontrolled confounding. Similarly, studies with incomplete or duplicate data were removed to ensure data integrity and avoid repetition. Finally, works with a high risk of bias as assessed by the MINORS scale were excluded in order to protect the validity and strength of conclusions drawn in the meta-analysis

### 2.2. Information Sources

PubMed, EMBASE, Scopus, and Cochrane Collaboration Library databases were used to collect studies between May and August 2023. No language restrictions or date limits were applied in this study. The references of the included studies were manually searched to identify potential studies that met the inclusion criteria.

### 2.3. Search Methods for Identification of Studies

The search terms used were ankle fractures, functional outcomes, quality of life, patient-reported outcome measures, predictors, and risk factors. MeSH terms and controlled vocabulary were used to broaden the search. Two independent reviewers conducted searches and screened the studies for eligibility. In the event of any disagreement, consensus was reached through discussion.

### 2.4. Data Extraction and Data Items

Regarding data extraction, two authors independently reviewed the studies, and a third author was consulted in case of disagreement to achieve consensus. The baseline characteristics of each study, such as study name, period, follow-up, region, study type, number of patients, number of females, age, fracture type, and treatment, were recorded. The main outcomes of interest were QoL, functionality, and pain. The modifiable predictive factors, including body mass index, smoking habit, diabetes, and treatment received, were defined according to standard diagnostic criteria or classifications. The mean value was directly obtained for body mass index. Smoking status refers to current smokers. Diabetes was defined as a type 1 or type 2 diagnosis, and most studies did not include a definition. Treatment was defined as open reduction with internal fixation or closed treatment. The non-modifiable factors were sex (male or female), mean age, and syndesmotic involvement. Syndesmosis impairment was defined in two ways: first, by infrasyndesmotic (Weber A) and trans-supra-syndesmotic (Weber B and C), and second, by the absence of lower tibiofibular ligament lesions, partial lesions, and complete lesions.

Due to the variety of QoL scales used in the original studies, the scales were divided according to their direction. The first group consisted of scales in which lower values indicated a worse score: Lower Extremity Functional Scale (LEFS), Global Perceived Effect (GPE), patient-reported outcome measurement system-physical function (PROMIS-PF), Short Form 36 score (SF-36), and Olerud and Molander Ankle Score (OMAS). The second group consisted of scales in which lower values indicated a better score: The Foot Function Index (FFI), self-rated foot and ankle questionnaire (SEFS), Visual Analog Scale (VAS), American Orthopaedic Foot and Ankle Society (AOFAS), Short Musculoskeletal Function Assessment (SMFA), and Timed Up and Go (TUG).

Additionally, the minimally clinically important difference was recorded for all scales that had clear evidence: 12 points for LEFS, 7.84 for PROMIS-PF, 4.1 for SF-36, 7.5 to 11.4 OMAS, 7 for SMFA, and 3.4 for TUG [10,11,12,13,14,15].

### 2.5. Assessment of Risk of Bias in Included Studies

Two authors independently evaluated the quality of the included studies using the Methodological Index for Non-Randomized Studies (MINORS) criteria, as shown in Table 1 [16].

### 2.6. Assessment of Results

To combine the quality of life scales in a standardized manner, the scales were divided into two groupings based on the direction of the values indicated by each scale: scales where lower values signified a poorer score and those where lower values signified a superior score. Due to the diverse scales employed and to regulate for heterogeneity, standardized mean differences (SMD) with a 95% confidence interval (CI) were utilized for dichotomous variables. Additionally, studies that reported outcomes in the form of correlations (beta) were combined by employing generic inverse variance. Meta-analysis was conducted using Review Manager 5.4, software provided by the Cochrane Collaboration. Heterogeneity was evaluated using both the chi-square test and I^2^ statistic, with I^2^ values ranging from 0 to 100% deemed indicative of low, moderate, and high heterogeneity at values of 25%, 50%, and 75%, respectively. A fixed-effects model was implemented when there was no statistical evidence of heterogeneity. Missing data were handled according to the guidelines stipulated in the Cochrane Handbook [17]. This standardized approach to aggregating and synthesizing the various quality of life scales across the included studies aimed to facilitate a rigorous comparative assessment.

**Table 1:** Assessment of the quality of studies through Methodological Index for Non-Randomized Studies (MINORS).

Study	Aim	Consecutive	Prospective	Endpoints	Assessment	Follow-Up	Loss	Study Size	Control	Contemporary	Baseline	Statistics	MINORS
Audet et al., 2021 [6]	2	2	0	2	2	2	0	2	1	2	1	2	17
De las Heras et al., 2016 [8]	2	2	0	2	2	2	2	2	1	2	0	2	19
Dean et al., 2017 [18]	2	2	0	1	1	2	0	2	1	2	0	2	15
Hancock et al., 2005 [19]	2	2	0	2	1	1	0	1	1	2	0	2	14
Keene et al., 2019 [20]	2	2	0	2	2	0	0	2	2	2	1	2	17
Park et al., 2019 [21]	2	2	0	2	2	0	0	1	2	2	2	2	17
Stavem et al., 2017 [22]	2	2	0	2	2	2	2	2	2	2	1	2	21
Simske et al., 2020 [23]	2	2	0	2	2	2	0	2	1	2	1	2	18

### 2.7. Risk of Bias across the Studies

Publication bias was assessed by visually inspecting the funnel plot for asymmetry, indicating possible publication bias. Review Manager 5.4 was the software used for this analysis.

### 2.8. Additional Analyses

A sensitivity analysis was conducted using the Review Manager 5.4. The sensitivity analysis involved removing the study with the highest weight from comparisons of all outcomes during the main analysis.

## 3. Results

### 3.1. Study Selection

**Figure 1:**
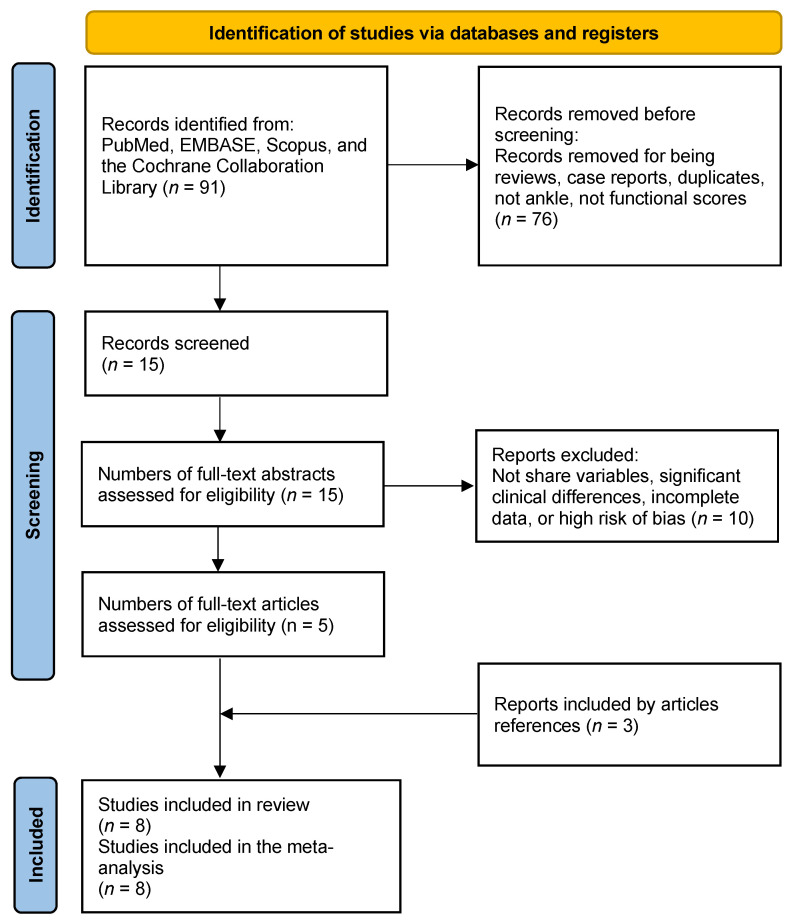
PRISMA flow diagram of the literature search results.

A comprehensive search of electronic databases yielded 91 citations. After a thorough review of the titles and abstracts, 76 studies were excluded due to non-compliance with the inclusion criteria. Subsequently, the full texts of the remaining 15 citations were examined in detail, ultimately leading to the exclusion of ten studies. Additional three studies meeting the inclusion criteria were identified through a reference search of the five remaining articles, resulting in a total of eight studies being included in the meta-analysis (refer to Figure 1) [6,8,18,19,20,21,22,23].

### 3.2. Study Quality and Study Characteristics

The methodological quality of the included studies was generally high, with six out of eight studies rated as high quality and two as fair quality (Table 1). However, several studies did not report the key elements of their methodology, which introduces some uncertainty. Specifically, studies failed to report whether data collection was performed prospectively. Some studies also mixed details on patients’ baseline characteristics and failed to account for all patients over the course of follow-up, reporting losses to follow-up. Overall, the methodological rigor was acceptable but could be strengthened by more transparently describing study protocols, especially related to data collection timing, baseline comparisons, and accounting for all enrolled participants.

Table 2 presents the key characteristics of the studies included. Eight studies with 2486 patients were included in the meta-analysis. Seven studies were retrospective cohort studies, and one was a case–control study. The mean age ranged from 46.7 to 70.0 years. In addition, 1510 (60.7%) patients were female. Five studies exclusively focused on surgically treated fractures, one study examined conservatively treated fractures, and two studies combined both treatment approaches.

### 3.3. Outcomes

**Figure 2:**
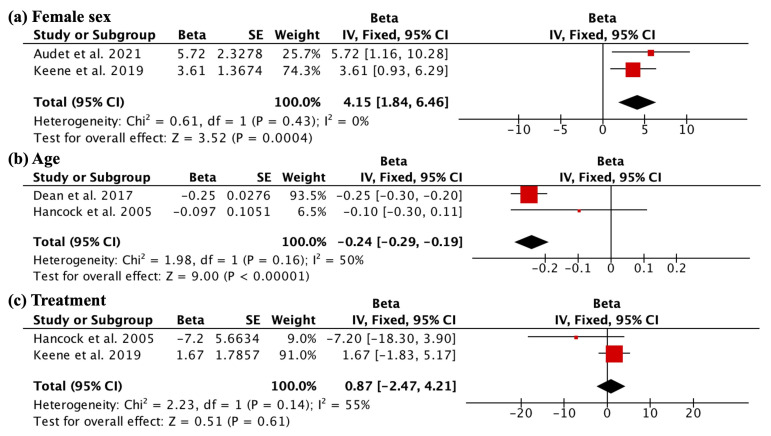
Influence of non-modifiable factors on functional outcomes. The first finding (**a**) reveals a correlation between female sex and worse scores on SMFA and TUG (beta 4.15, 95% CI 1.84 to 6.46; studies = 2). Advanced age is also associated with worse outcomes, as shown in (**b**), where a negative correlation is observed between age and outcomes on LEFS and PROMIS-PF (beta −0.24, 95% CI −0.29 to −0.19; studies = 2). However, in (**c**), no correlation is observed between treatment and the observed scores (beta 0.87, 95% CI −2.47 to 4.21; studies = 2).

Regarding non-modifiable factors, sex, as assessed by SMFA and TUG, showed a correlation between female sex and worse scores (beta 4.15, 95% CI 1.84 to 6.46; studies = 2) (Figure 2a). However, this correlation did not exceed the MCID difference for the SMFA or TUG tests. Keene et al. included both conservatively and surgically treated studies, while Audet et al. included only surgically treated patients; both studies showed individually worse outcomes in female patients. Age, as assessed by LEFS and PROMIS-PF, demonstrated a correlation between advanced age and worse outcomes (beta −0.24, 95% CI −0.29 to −0.19; studies = 2) (Figure 2b). Regarding age, the Dean et al. study included surgical patients, and age showed significantly worse outcomes, whereas Hancock et al. included conservatively treated ankle fracture patients and showed no significant correlation between older age and worse outcomes. Regarding treatment, as assessed by OMAS, there was no correlation observed with the observed scores (beta 0.87, 95% CI −2.47 to 4.21; studies = 2) (Figure 2c). In this case, Hancock et al. included conservatively treated patients, and Keene et al. included both conservatively and surgically treated patients. None of the included studies showed any significant correlation.

**Figure 3:**
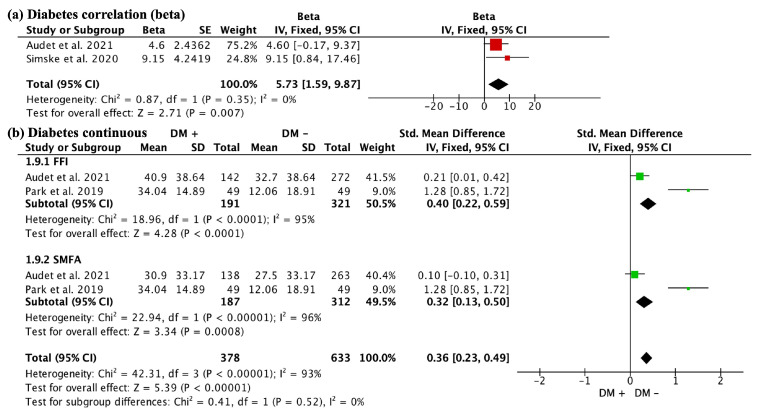
Impact of diabetes on functional outcomes. The first finding (**a**) reveals a correlation between diabetes and worse outcomes on FFI (beta 5.73, 95% CI 1.59 to 9.87; studies = 2). Additionally, when analyzing diabetes together with metabolic syndrome, worse outcomes are generally observed (**b**). DM+: patient with diabetes; DM−: patient without diabetes; FFI: Foot Function Index; SMFA: Short Musculoskeletal Function Assessment.

When analyzing diabetes, as assessed by FFI, a correlation was found with worse outcomes (beta 5.73, 95% CI 1.59 to 9.87; studies = 2) (Figure 3a). When analyzing diabetes together with metabolic syndrome, worse outcomes were generally observed (SMD 0.36, 95% CI 0.23 to 0.49; participants = 1011; studies = 4; I^2^ = 93%). When divided by the FFI and SMFA scales, patients with this condition also showed worse results ((SMD 0.40, 95% CI 0.22 to 0.59; participants = 512; studies = 2; I^2^ = 95%)) and (SMD (0.32, 95% CI 0.13 to 0.50; participants = 499; studies = 2; I^2^ = 96%) (Figure 3b). However, this did not exceed the MCID difference for SMFA, which was only assessable in the study by Audet et al. [6].

**Figure 4:**
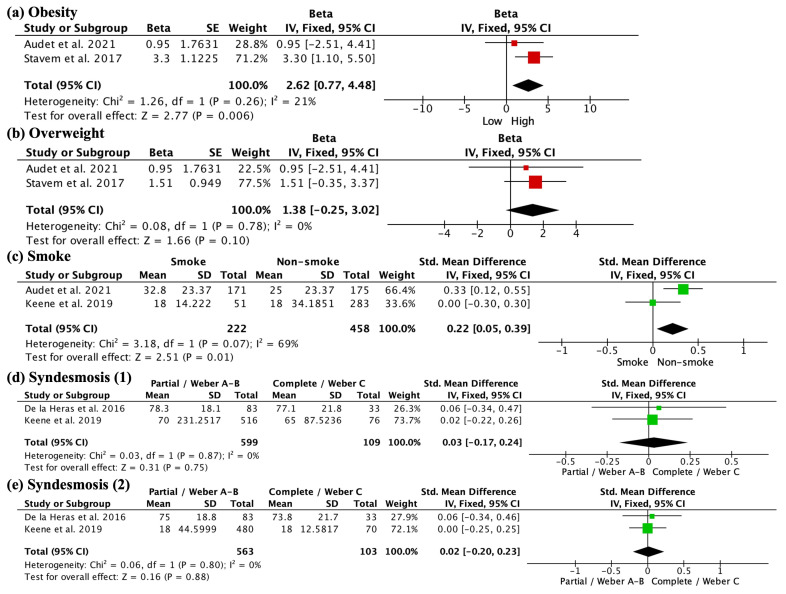
Influence of modifiable factors on functional outcomes. Firstly, high BMI and obesity are associated with worse scores on quality of life scales, indicating a negative impact (**a**). However, no significant correlation is found between being overweight and functional scores (**b**). Additionally, smokers present greater disability in functional outcomes (**c**), suggesting a detrimental effect of smoking. On the other hand, no significant differences are observed in syndesmosis involvement when assessed by OMAS, SF-36, TUG, or AOFAS (**d**,**e**). Syndesmosis (1) included OMAS and SF-36 scales. Syndesmosis (2) included TUG or AOFAS scales.

Regarding modifiable risk factors, BMI was assessed using the FFI and SEFS. High BMI and obesity were correlated with worse scores on the quality of life scales (beta 2.62, 95% CI 0.77 to 4.48; studies = 2) (Figure 4a). No correlation was found when overweight was used (beta = 1.38, 95% CI −0.25 to 3.02; studies = 2) (Figure 4b). Regarding smoking, as assessed by the SMFA and TUG, smokers presented greater disability in functional outcomes (SMD 0.22, 95% CI 0.05 to 0.39; participants = 680; studies = 2; I^2^ = 69%) (Figure 4c). The study by Keene et al., which included both conservatively and surgically treated patients, showed no significant differences. The SMFA in the study by Audet et al. exceeded the MCID (11). Finally, regarding syndesmosis involvement, no significant differences were found between the affected and unaffected patients, as assessed by OMAS and SF-36 (syndesmosis 1) (SMD 0.03, 95% CI −0.17 to 0.24; participants = 708; studies = 2; I^2^ = 0%) (Figure 4d), or in terms of assessment with TUG or AOFAS (syndesmosis 2) (SMD 0.02, 95% CI −0.20, 0.23; participants = 666; studies = 2; I^2^ = 0%) (Figure 4e). None of the studies analyzed individually found significant correlations, as De las Heras et al. included surgically treated patients, while Keene et al. included surgical and conservative treatment.

### 3.4. Additional Analyses

No sensitivity analysis or publication bias assessment was conducted due to the small number of studies included in the effect sizes.

## 4. Discussion

This meta-analysis observed that female sex, advanced age, diabetes or metabolic syndrome, high BMI, and smoking were correlated with worse outcomes in the quality of life and functional disability scales.

A significant correlation was found between advanced age and worse functional outcomes in the quality of life and functional disability scales [24]. This may be related to the development of accelerated osteoarthritis, as nearly 40% of patients treated with open reduction and internal fixation (ORIF) for ankle fractures develop osteoarthritis 18 years post-treatment [25,26,27]. Women had worse functional outcomes than men [24]. This could be related to estrogen levels [28]. Diabetes and metabolic syndrome are correlated with worse functional outcomes after ankle fractures [24]. It is important to note that, in general, risk factors such as diabetes, obesity, and smoking have primarily been studied in relation to radiological outcomes such as non-union [25,26,27]. A correlation was found between a high BMI and poor functional outcomes.

Smoking was also correlated with worse functional outcomes, which is logical because of the physiological effects of tobacco on bone and fracture healing [28]. Smoking has a transient effect on the tissue microenvironment and prolongs inflammatory and reparative cellular functions, leading to delayed healing and complications [29]. Smoking cessation rapidly restored the tissue microenvironment and inflammatory cellular functions within 4 weeks, but the proliferative response remained deficient. Nicotine and nicotine replacement medications appear to attenuate inflammation and improve proliferation; however, their effects are marginal [29]. Additionally, Audet et al. (2022) observed that current smokers were at a higher risk than former smokers [7]. These unfavorable results could lead to the idea of smoking cessation as an inclusion criterion in surgery or pre- or postoperative cessation therapies [30]. However, it is difficult to do so in young patients because of the risk factors that increase susceptibility to smoking [30]. These results are in line with those of previous meta-analyses on spine surgery or hip arthroplasty [31,32]. Future studies should compare patients who continue smoking with those who quit smoking. Previous studies unrelated to ankle fractures have reported that among patients with back pain, smoking status was associated with greater reported pain levels [33].

Syndesmotic involvement is associated with poor functional outcomes [34]. Precision in syndesmotic reduction is crucial for obtaining good functional outcomes and avoiding future complications. The exact fracture type could not be compared in this study owing to the absence of information and comparability.

Several strategies can be implemented to enhance outcomes. Smoking cessation programs or nicotine replacement therapy should be initiated prior to surgery and supported by resources and counseling to assist patients in quitting smoking before and after the procedure [35]. Clinical protocols should be developed to identify high-risk smokers and provide them with more intensive interventions. Medications such as nicotine and varenicline should be explored as potential options to reduce complications in smokers undergoing surgery [36].

The MCID could not be consistently evaluated because most studies were compared in the form of correlations. In this study, purely radiological factors could not be compared despite their influence [8]. Another interesting variable collected in the study by Hancock et al. was dorsiflexion, a measurable parameter in clinical observations that showed better initial dorsiflexion results in better outcomes [19]. This is interesting because it could limit mobility and, therefore, affect long-term functional outcomes, although possible confounding factors such as edema, rehabilitation, or physical activity should be considered. This is because functional outcomes are related to the baseline state, as highlighted by Simske et al., where the final outcomes depend in part on the baseline state [23]. Another important point that was not analyzed was the influence of psychiatric factors, which could not be analyzed because of the small number of studies included. However, a relationship between metabolic syndrome and depression has also been described [37].

However, it is important to consider the inclusion criteria of these studies. Hancock et al. included conservatively treated ankle fractures, and Keene et al. included both conservatively and surgically treated ankle fractures [18,19,20]. The remaining studies only included conservatively treated fractures. When visually analyzing the influence of these characteristics, it was observed that these studies showed less correlation with many of the analyzed variables. For example, Hancock et al. did not observe any correlation with worse functional outcomes [19]. Keene et al. also observed less correlation with sex, and although syndesmotic involvement did not show differences, this study showed less correlation when assessed by beta [20]. Finally, the influence of treatment on functional outcomes did not show significant differences, although these results were only evaluated by the Hancock et al. and Keene et al. studies [18,19,20]. Hancock et al. treated patients conservatively, while Keene et al. included both surgical and non-surgical treatment groups but did not report outcomes separately. None of the studies found a significant correlation when analyzed individually. Therefore, the influence of including mixed treatment approaches (surgical and conservative) versus exclusively surgically treated patients on patient-reported outcomes remains unclear. As no significant associations were found in single studies, the impact that mixing treatment modalities may have had on outcomes is unclear. Reporting separated by intervention is necessary to fully understand any relationships without potential confounding from heterogeneous management strategies within the studies. These observations suggest that the influence of all these factors, including age, sex, smoking, obesity, and diabetes, mainly predicts the outcomes of surgical treatment. This is logical, as this type of fracture presents greater aggression, both due to the fracture itself and the additional aggression that surgery entails. In requiring a greater energy supply for this type of fracture and management with altered circulation or microcirculation, such as smoking, diabetes, obesity, or worse functioning of systems with age, functional outcomes will be worse.

### Limitations and Strengths

This study has several limitations that could influence the rigor and completeness of the analyses. A small number of articles were included in the subgroup comparisons, regression analyses, and sensitivity analyses, restricting the ability to perform more detailed assessments. Some studies did not control for potential confounding factors, and socioeconomic and psychiatric metrics that may impact outcomes were not examined. Scales were amalgamated using standardized mean differences despite possible heterogeneity, and missing data were handled using the Cochrane calculator, which could affect validity. Patient numbers at follow-up were, in some cases, unspecified, such as with respect to former versus never smokers. Diabetes analyses encompassed insulin resistance without clarifying whether altered results from this were excluded. However, removing the diabetes study did not transform this directionality. A digitizing tool was used in one study, potentially impacting the precision. While other eligible studies evaluated relevant factors, they primarily reported demographics rather than quality of life, so they were excluded. Presenting correlations or continuous variables depends on the source data format. Overall, this research had constraints that may have influenced the findings to an uncertain degree, suggesting prospects for enhancing rigor in future related investigations. Some studies also included patients treated both surgically and conservatively without separating the outcomes for each group. This made the interpretation of results more difficult and increased heterogeneity. Reporting combined outcomes without stratification by treatment modality (surgical versus non-surgical) confounds interpretation. Nor was it divided by fracture type or trauma type (low/high energy). On the positive side, the strength was the global focus on specific outcome influences and identifying limitations that can inform improved future study designs, as well as recognizing at-risk patients for enhanced follow-up and treatment.

## 5. Conclusions

The risk factors and patient characteristics that can influence functional outcomes after ankle fractures include age, sex, presence of diabetes or metabolic syndrome, high BMI, and smoking. Future studies should aim to enroll more homogenous patient populations in terms of the treatment approach by clearly reporting outcomes separately for surgically versus conservatively treated groups. Limiting inclusion criteria to specific, comparable fracture types rather than broad “ankle fractures” would help reduce variability. Researchers should also explicitly describe inclusion/exclusion criteria, treatment protocols, patient demographics, clinical factors, and characteristics to improve transparency and reproducibility.

## Figures and Tables

**Table 2:** Baseline characteristics of the eight included studies.

Study	Period	Region	Type of Study	*n*	Female	Age	Fracture Type	Treatment
Audet et al., 2021 [6]	2006 to 2015	USA	Retrospective study	416	224	46.7	OTA/AO 44A, 44B, or 44C	Surgery
De las Heras et al., 2016 [8]	2006 to 2017	Spain	Retrospective study	266	145	48.6	AO/OTA 4 type 44	Surgery
Dean et al., 2017 [18]	2001 to 2013	USA	Retrospective study	142	64	52.7	AO/OTA 44-A 44-B 44-C	Surgery
Hancock et al., 2005 [19]	2001 to 2003	Australia	Retrospective study	64	30	49.0	NS	Conservative
Keene et al., 2019 [20]	2004 to 2010	UK	Retrospective study	592	441	70.0	NS	Surgery/Conservative
Park et al., 2019 [21]	2013 to 2016	South Korea	Case–control study	98	52	59.2	SER II SER III SER IV	Surgery
Stavem et al., 2017 [22]	2009 to 2011	Norway	Retrospective study	479	277	52.7	Weber A, B and C	Surgery
Simske et al., 2020 [23]	2006 to 2015	USA	Retrospective study	429	267	NS	AO/OTA 44A–C	Surgery/Conservative

NS: not specified; SER: supination external rotation.

## Data Availability

No new data were created or analyzed in this study. Data sharing is not applicable to this article.
